# Supplementary material for: Estrogen receptor beta in astrocytes modulates cognitive function in mid-age female mice
Source: Nat Commun. 2023 Sep 28;14:6044. doi: 10.1038/s41467-023-41723-7 (PMC10533869; doi:10.1038/s41467-023-41723-7)
Supplement: Supplementary file 1 — Supplementary Information [file 41467_2023_41723_MOESM1_ESM.pdf]

Supplemental Materials

Supplemental Figures and Legends

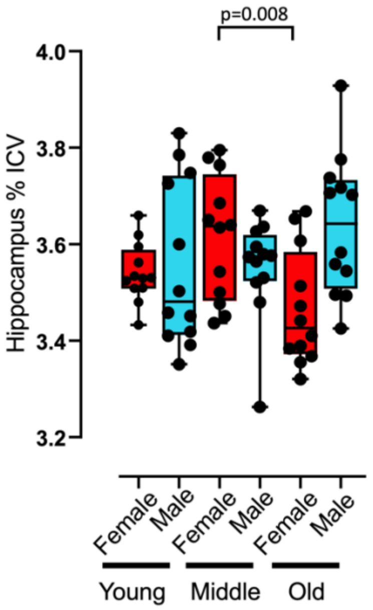

Supplemental Figure 1. Sham surgery treated female, but not male, mice showed hippocampal atrophy at midlife compared to old age. Substructure volumes, assessed by MRI, taken as a percentage of intercranial volume (ICV) are shown for whole hippocampus n=12 mice per group. P values were calculated by two-sided Welch's t-test. All box plots with centre lines showing the medians, boxes indicating the interquartile range, and whiskers indicating from the minimum and to the maximum values.

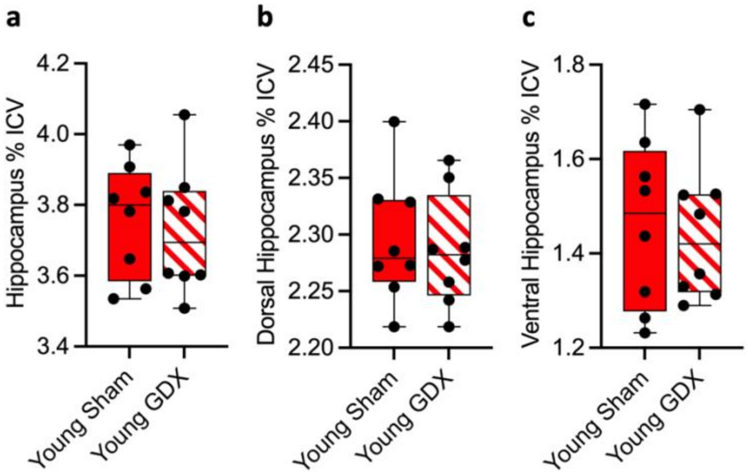

Supplemental Figure 2. Gonadectomy did not induce hippocampal atrophy at young age. Substructure volumes, assessed by MRI, taken as a percentage of intercranial volume (ICV) are shown for **(a)** hippocampus, **(b)** dorsal hippocampus, and **(c)** ventral hippocampus. There were no differences in substructure volumes between groups at young age. separate, independent imaging analysis experiment, n=8 mice per group. P values were calculated by two-sided Welch's t-test. All box plots with centre lines showing the medians, boxes indicating the interquartile range, and whiskers indicating from the minimum and to the maximum values.

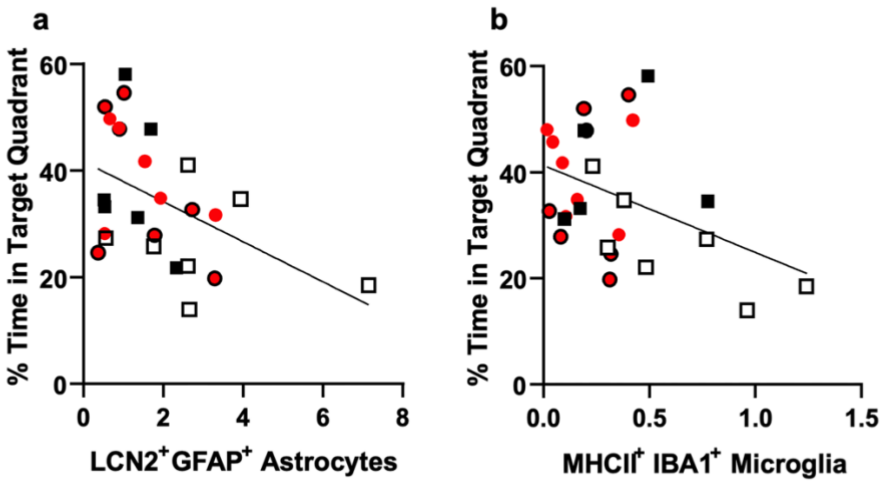

Supplemental Figure 3. Correlation of MWM cognitive performance with glia activation.

a-b) % Time in target quadrant is negatively correlated with **a)** LNC2^{+}GFAP^{+} astrocytes (r=-0.46203, p=0.017) and **b)** MHCII^{+} IBA1^{+} microglia (r=-0.4151, p=0.0350). red dot, Young Sham; red dot with black rim, Young GDX; black square, Midlife Sham; white square, Midlife GDX females. Pearson correlation analyses.

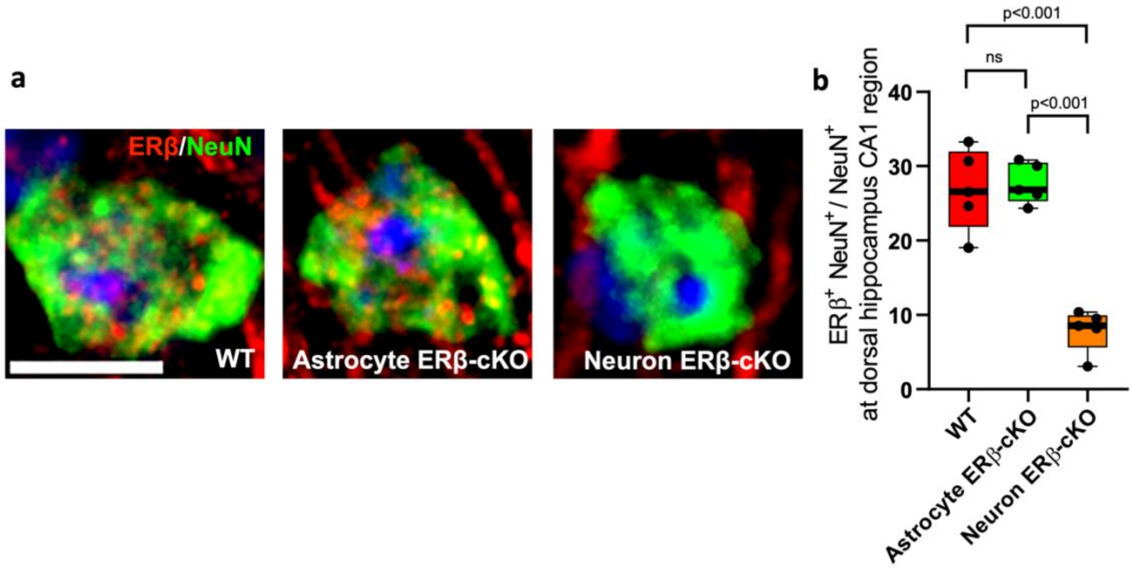

Supplemental Figure 4. ERβ expression remained intact in neurons of the astrocyte-ERβ cKO. (a) Representative 40x image of ERβ (red) and NeuN (green) with colocalization (yellow) in dorsal hippocampus CA1 region from WT littermates (WT, left), *mGFAP-Cre(77.6); ERβ*$^{fl/fl}$ (astrocyte ERβ cKO, middle); and *rNSE-Cre; ERβ*$^{fl/fl}$ (neuron ERβ cKO, right) mice. Bar= 10 um. **(b)** Quantitative analysis of area fraction of colocalized ERβ^{+} NueN^{+} in dorsal hippocampal CA1 region from WT littermates (WT, red), astrocyte ERβ cKO (green), and *rNSE-Cre; ERβ*$^{fl/fl}$ (neuron ERβ cKO, orange) mice. A significant decrease of ERβ^{+} NeuN^{+} colocalized area was observed in neuron ERβ cKO compared to WT littermates, while there was no decrease in ERβ^{+} NeuN^{+} colocalized area in astrocyte ERβ cKO. n=5 per group. P values were calculated by two-sided Mann Whitney *U* test All box plots with centre lines showing the medians, boxes indicating the interquartile range, and whiskers indicating from the minimum and to the maximum values.

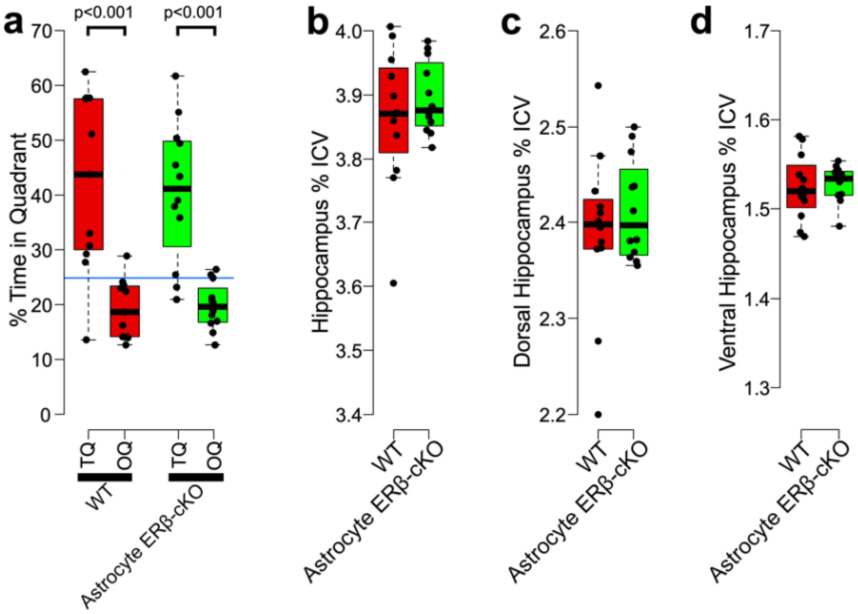

Supplemental Figure 5. Selective deletion of ERβ from astrocytes does not induce cognitive impairment or hippocampal atrophy in young female mice. (a) Wild type and conditional knockouts of ERβ in astrocytes (astrocyte ERβ cKO) were evaluated at young age for spatial reference memory by MWM. Both WT and astrocyte ERβ cKO showed intact spatial reference memory at young age. Blue line indicates the null hypothesis (25% in TQ). WT n=11, astrocyte ERβ cKO n=12. Substructure volumes, assessed by MRI, taken as a percentage of intercranial volume (ICV) are shown for **(b)** hippocampus, **(c)** dorsal hippocampus, and **(d)** ventral hippocampus. N=12 mice per group. There was no difference in substructure volumes between groups at young age. P values were calculated by either two-sided Mann Whitney *U* test (a) or two-sided Welch's t-test (b-d). All box plots with centre lines showing the medians, boxes indicating the interquartile range, and whiskers indicating from the minimum and to the maximum values.
