# Supplementary material for: Estrogen receptor beta in astrocytes modulates cognitive function in mid-age female mice
Source: Nat Commun. 2023 Sep 28;14:6044. doi: 10.1038/s41467-023-41723-7 (PMC10533869; doi:10.1038/s41467-023-41723-7)
Supplement: Supplementary file 3 — Reporting Summary [file 41467_2023_41723_MOESM3_ESM.pdf]

nature portfolio

Corresponding author(s): Rhonda Voskuhl

Last updated by author(s): Aug 22, 2023

Reporting Summary

Nature Portfolio wishes to improve the reproducibility of the work that we publish. This form provides structure for consistency and transparency in reporting. For further information on Nature Portfolio policies, see our Editorial Policies and the Editorial Policy Checklist.

Statistics

For all statistical analyses, confirm that the following items are present in the figure legend, table legend, main text, or Methods section.

n/a	Confirmed	
☐	☒	The exact sample size (*n*) for each experimental group/condition, given as a discrete number and unit of measurement
☐	☒	A statement on whether measurements were taken from distinct samples or whether the same sample was measured repeatedly
☐	☒	The statistical test(s) used AND whether they are one- or two-sided<br>*Only common tests should be described solely by name; describe more complex techniques in the Methods section.*
☐	☒	A description of all covariates tested
☐	☒	A description of any assumptions or corrections, such as tests of normality and adjustment for multiple comparisons
☐	☒	A full description of the statistical parameters including central tendency (e.g. means) or other basic estimates (e.g. regression coefficient) AND variation (e.g. standard deviation) or associated estimates of uncertainty (e.g. confidence intervals)
☐	☒	For null hypothesis testing, the test statistic (e.g. *F*, *t*, *r*) with confidence intervals, effect sizes, degrees of freedom and *P* value noted<br>*Give P values as exact values whenever suitable.*
☒	☐	For Bayesian analysis, information on the choice of priors and Markov chain Monte Carlo settings
☒	☐	For hierarchical and complex designs, identification of the appropriate level for tests and full reporting of outcomes
☐	☒	Estimates of effect sizes (e.g. Cohen's *d*, Pearson's *r*), indicating how they were calculated

Our web collection on statistics for biologists contains articles on many of the points above.

Software and code

Policy information about availability of computer code

Data collection	TopScan (Clever Sys, Inc., Reston, VA) for Morris Water Maze. cellSens 4.0 (Olympus America Inc.) for confocal imaging.<br>No custom codes or algorithms were used to generate results.
Data analysis	Prism 9.5.0 for the analysis of behavioral studies, and histology. NIH Image J (1.54) Software for image analysis of immunostaining. R (https://www.r-project.org) for the analysis on atlas-based morphometry and RNA-Seq results.<br>No custom codes or algorithms were used to generate results.

For manuscripts utilizing custom algorithms or software that are central to the research but not yet described in published literature, software must be made available to editors and reviewers. We strongly encourage code deposition in a community repository (e.g. GitHub). See the Nature Portfolio guidelines for submitting code & software for further information.

Data

Policy information about availability of data

All manuscripts must include a data availability statement. This statement should provide the following information, where applicable:

- Accession codes, unique identifiers, or web links for publicly available datasets
- A description of any restrictions on data availability
- For clinical datasets or third party data, please ensure that the statement adheres to our policy

Datasets of RNA-sequencing generated during this study are available in the GEO (Gene Expression Omnibus; www.ncbi.nlm.nih.gov/geo/) under the accession

number GSE220288.
The data that support this study are available within the article and its Supplementary Information files. Source data are provided with this paper.

Human research participants

Policy information about studies involving human research participants and Sex and Gender in Research.

Reporting on sex and gender	N/A
Population characteristics	N/A
Recruitment	N/A
Ethics oversight	N/A

Note that full information on the approval of the study protocol must also be provided in the manuscript.

Field-specific reporting

Please select the one below that is the best fit for your research. If you are not sure, read the appropriate sections before making your selection.

☒ Life sciences ☐ Behavioural & social sciences ☐ Ecological, evolutionary & environmental sciences

For a reference copy of the document with all sections, see nature.com/documents/nr-reporting-summary-flat.pdf

Life sciences study design

All studies must disclose on these points even when the disclosure is negative.

Sample size	Sample sizes were based on previous publications from our laboratory and others considering expected biologic differences in the context of observed variability of each outcome, as well as numbers that are logistically feasible to manage with rigor. MRI for regional brain atrophy (Meyer et al, NeuroImage, 2017), behavioral cognitive testing (Harburger et al, Behav Brain Res., 2007, Wang et al, Nat Neurosci. 2020), pathology (Ziehn et al, Lab Invest. 2010, Rodríguez et al, Neurobiol Aging. 2014), treatment (Mayer et al, Lab Invest. 2023, Frick et al, Neuroscience, 2002), RNA-Seq of astrocytes (Itoh et al, PNAS, 2018).
Data exclusions	No data were excluded from the data, with the exception of damaged tissues during immunostaining.
Replication	All behavioral experiments described were replicated successfully in at least two independent cohorts of animals. The ERβ ligand treatment study was successfully conducted twice using biologically independent animals.
Randomization	For all experiments involving transgenic animals, littermates were randomly assigned to experimental groups. For all experiments involving C57BL/6J wildtype mice, age and sex were identical throughout cohorts and cagemates were randomly assigned to experimental groups.
Blinding	All experiments were performed and analyzed in a blinded manner.

Reporting for specific materials, systems and methods

We require information from authors about some types of materials, experimental systems and methods used in many studies. Here, indicate whether each material, system or method listed is relevant to your study. If you are not sure if a list item applies to your research, read the appropriate section before selecting a response.

Materials & experimental systems

n/a	Involved in the study	
☐	☒	Antibodies
☒	☐	Eukaryotic cell lines
☒	☐	Palaeontology and archaeology
☐	☒	Animals and other organisms
☒	☐	Clinical data
☒	☐	Dual use research of concern

Methods

n/a	Involved in the study	
☒	☐	ChIP-seq
☒	☐	Flow cytometry
☐	☒	MRI-based neuroimaging

Antibodies

Antibodies used	The following primary antibodies were used: Goat anti- mouse Lipocalin-2 (LGN2) Antibody (at 1:50, R&D, cat#AF1857), Rat anti-GFAP (at 1:500, Thermo Fisher, cat# 13-0300, clone 2.2B10), Rabbit anti-Iba1 (at 1:1000, Wako Chemicals USA Inc. cat# 019-19741), Rat anti-MHCII (at 1:500, Biolegend, cat#107602, clone M5/114.15.2), Rabbit anti-P2RY12 (at 1:1000, AnaSpec, cat#AS-55043A), Rat anti-CLEC7A (at 1:200, InvivoGen, cat#mabg-mdect, clone R1-8g7), Rat anti-P2RY12 (at 1:100, Biolegend, cat#848002, clone S16007D), Rabbit anti-CLEC7A (at 1:200, Thermo Fisher, cat#PA-534382), Rabbit anti-Synapsin 1 (at 1:500 dilution, Synaptic Systems, cat#106 103), Guinea pig anti-PSD95 (at 1:250 dilution, Synaptic Systems, cat#124 014), Mouse anti-HA (at 1:500, Biolegend, cat#901522, clone 16B12), Rabbit anti-Mouse ALDH1L1 (at 1:250, Abcam, cat#ab87117), Mouse anti-CC1 (at 1:500, Millipore, cat#OP80, clone CC-1), Rabbit anti-mouse ERβ (at 1:100, Thermo Fisher, cat#PA1310B), Guinea pig anti-NeuN (at 1:500 dilution, Synaptic Systems, cat#266 004). The following secondary antibodies were used at 1:500 dilution for staining the tissues: Goat anti-rabbit- Alexa Fluor Plus 647 (cat#A32733, Thermo Fisher), goat anti-Guinea Pig - DyLight 550 (cat# SA5-10095, Thermo Fisher), goat anti-rat- Alexa Fluor488 (Cat# 112-545-167, Jackson Immunoresearch), and goat anti-rat-Cy3 (cat#112-165-167, Jackson Immunoresearch), Goat anti-rat Cy5 (cat# 112-175-167, Jackson Immunoresearch), Goat anti-rabbit TRITC (cat# 111-025-144 , Jackson Immunoresearch), Donkey anti-goat Cy5 (Ab#6566, Abcam), Donkey Anti-rat Cy3 (cat#712-165-153, Jackson Immunoresearch), Goat anti-mouse- Cy3 (cat#115-545-166, Jackson Immunoresearch).
Validation	All antibodies used in this manuscript were sourced from vendors and have previously been confirmed by authors for their applicability in the present study. The validation of these antibodies can be found in peer reviewed publications by us and others, and/or vendors' website.<br>Goat anti- mouse Lipocalin-2 (LGN2) Antibody (at 1:50, R&D, cat#AF1857) was validated and used in multiple publications (e.g. Tassoni et al, Sci. Rep., 2019).<br>Rat anti-GFAP (at 1:500, Thermo Fisher, cat# 13-0300) was validated and used in multiple publications (e.g. Itoh et al, PNAS, 2017, Tassoni et al, Sci. Rep., 2019).<br>Rabbit anti-Iba1 (at 1:1000, Wako Chemicals USA Inc. cat# 019-19741) was validated and used in multiple publications (e.g. Itoh et al, PNAS, 2017, Meyer et al, Lab Inv., 2023).<br>Rat anti-MHCII (at 1:500, Biolegend, cat#107602) was validated and used in multiple publications (e.g. Voskuhl et al, PNAS, 2019, Meyer et al, Lab Inv., 2023).<br>Mouse anti-HA (at 1:500, Biolegend, cat#901522) was validated and used in multiple publications (e.g. Itoh et al, PNAS, 2017, Voskuhl et al, PNAS, 2019).<br>Rabbit anti-P2RY12 (at 1:1000, AnaSpec, cat#AS-55043A) was validated and used in multiple publications (e.g. Werneburg et al, Immunity, 2021).<br>Rat anti-CLEC7A (at 1:200, InvivoGen, cat#mabg-mdect) was validated and used in multiple publications (e.g. Werneburg et al, Immunity, 2021).<br>Rabbit anti-Synapsin 1 (at 1:500 dilution, Synaptic Systems, cat# 106 103) was validated by the company (https://sysy.com/product/106103) and used in multiple publications (e.g. Shi et al, Neuron., 2021).<br>Guinea pig anti-PSD95 (at 1:250 dilution, Synaptic Systems, cat#124 014) was previously used in multiple publications (e.g. Grabner et al, Nature Comm., 2023).<br>Rabbit anti-Mouse ALDH1L1 (at 1:250, Abcam, cat#ab87117) was previously used in multiple publications (e.g. Jiwaji et al, Nature Comm., 2022, Burda et al, Nature, 2022).<br>Mouse anti-CC1 (at 1:500, Millipore, cat#OP80) was validated and used in multiple publications (e.g. Voskuhl et al, PNAS, 2019, Meyer et al, Lab Inv., 2023).<br>Rabbit anti-mouse ERβ (at 1:100, Thermo Fisher, Cat: PA1310B) was previously validated and used in multiple publications (e.g. Kim et al, Sci. Rep. 2019).<br>Guinea pig anti-NeuN (at 1:500 dilution, Synaptic Systems, cat# 266 004) was validated by Synaptic Systems (https://sysy.com/product/266004) and used in multiple publications (e.g. Gangwani et al, Cell Rep., 2023).

Animals and other research organisms

Policy information about studies involving animals; ARRIVE guidelines recommended for reporting animal research, and Sex and Gender in Research

Laboratory animals	All mice used in this study were from the C57BL/6J background. Mice were evaluated at three ages: young (3-4 months), midlife (12-14 months), and old (20-22 months). We generated mice with selective deletion of ERβ in astrocytes. GFAP-Cre:ERβfl/fl (astrocyte ERβ cKO) were generated by crossing the mGFAP-Cre 77.6 line (B6.Cg-Tg(Gfap-Cre)77.6Mvs/2J, JAX) with the exon 3 ERβ fl/fl line. NSE-Cre:ERβfl/fl (neuron ERβ cKO) were also generated by crossing the rNSEII-Cre line with the exon 3 ERβfl/fl line. GFAP-Cre:RiboTag mice were generated by crossing RiboTag mice (B6J.129(Cg)-Rpl22tm1.1Psam/SjJ, Jackson Lab) with the mGFAP-Cre 77.6 line (B6.Cg-Tg(Gfap-Cre)77.6Mvs/2J, Jackson lab). To generate GFAP-Cre:RiboTag: ERβfl/fl mice, RiboTag mice (B6J.129(Cg)-Rpl22tm1.1Psam/SjJ, Jackson Lab) were crossed with GFAP-Cre: ERβfl/fl. All mice were housed in a facility with 12-h light/dark cycle in temperature- and humidity- controlled and were allowed free access to food and water.
Wild animals	This study did not involve wild animals.
Reporting on sex	Yes, sex is report and of focus in this paper.<br>A sex difference observed prompted further study
Field-collected samples	This study did not involve field-collected samples.
Ethics oversight	All procedures were done in accordance with the guidelines of the National Institutes of Health and the Chancellor's Animal Research Committee of the University of California, Los Angeles Office for the Protection of Research Subjects. . All animal experiments were

approved by the Chancellor's Animal Research Committee of the University of California, Los Angeles Office (ARC#1998-001).

Note that full information on the approval of the study protocol must also be provided in the manuscript.

Magnetic resonance imaging

Experimental design

Design type	Structural MRI
Design specifications	This study did not use design specifications.
Behavioral performance measures	This study did not use behavioral performance measures.

Acquisition

Imaging type(s)	structural
Field strength	7T
Sequence & imaging parameters	Rapid-acquisition with relaxation enhancement (RARE) sequence with the following parameters: TR/TEeff 3500/32 ms, ETL 16, matrix: 256 × 192 × 100, voxel dimensions: 100 × 100 × 100 µm3. Total imaging time was 93 minutes.
Area of acquisition	Brain

Diffusion MRI ☐ Used ☒ Not used

Preprocessing

Preprocessing software	BrainSuite 19b and SPMMouse, SPM8, and MATLAB R2013a
Normalization	Manual rigid body alignment, followed by linear registration with modulation, and lastly non-linear spatial normalization with modulation using DARTEL.
Normalization template	Mortimer Space Atlas. Briefly, an atlas comprising 30 adult female and 30 adult male mice. (Meyer 2017 NeuroImage)
Noise and artifact removal	Standard inhomogeneity correction in SPM.
Volume censoring	This study did not use volume censoring.

Statistical modeling & inference

Model type and settings	multivariate general linear model
Effect(s) tested	structure volume, age x sex interaction, GDX x age interaction, ANOVA design

Specify type of analysis: ☐ Whole brain ☒ ROI-based ☐ Both

Anatomical location(s)	Frontal cortex, striatum, whole hippocampus, dorsal hippocampus, and ventral hippocampus were manually delineated on the average template and then computationally warped out to individual images for analysis.
Statistic type for inference (See Eklund et al. 2016)	This study did not use cluster-wise methods.
Correction	FDR

Models & analysis

n/a	Involved in the study
☒	☐ Functional and/or effective connectivity
☒	☐ Graph analysis
☒	☐ Multivariate modeling or predictive analysis
